# Screening Candidate Genes Regulating Placental Development from Trophoblast Transcriptome at Early Pregnancy in Dazu Black Goats (*Capra hircus*)

**DOI:** 10.3390/ani11072132

**Published:** 2021-07-19

**Authors:** Nanjian Luo, Wenqiang Cheng, Yumei Zhou, Bowen Gu, Zhongquan Zhao, Yongju Zhao

**Affiliations:** College of Animal Science and Technology, Southwest University, Chongqing 400715, China; luonanjian28@sina.cn (N.L.); c_wqiang@163.com (W.C.); zym1998@email.swu.edu.cn (Y.Z.); m17783249677@163.com (B.G.); zhaozhongquan@swu.edu.cn (Z.Z.)

**Keywords:** trophoblast, transcriptome, early pregnancy, placenta development, goat

## Abstract

**Simple Summary:**

The trophoblast is an original placental tissue whose normal proliferation, differentiation, migration, adhesion, and angiopoiesis are essential for placenta formation and fetal survival during early pregnancy. However, the key genes and molecular mechanisms involved in placenta development in goats are unknown. Herein, the morphology and histological structures of trophoblast tissues from day 20 to 30 of pregnancy were determined. RNA-sequencing was used to screen potential functional genes in common highly expressed and differentially expressed genes. RAP1 signaling pathway was used as the contact center and coordinated with other pathways to regulate placenta development. This study could provide insights into the molecular mechanisms underlying ruminant placentation.

**Abstract:**

This study explored the trophoblast transcriptome to understand potential functional genes involved in early placental development in goats and their enriched signaling pathways. Trophoblast samples were collected from nine Dazu Black goats on days 20, 25, and 30 of pregnancy (D20, D25, and D30). As the pregnancy progressed, the morphology and histological structures showed significant growth, adhesion, and angiogenesis. A total of 23,253 commonly expressed genes (CEGs) and 4439 differently expressed genes (DEGs) were detected by RNA sequencing. The common highly expressed genes (ChEGs) (the top 100 CEGs) with the highest FPKM percentage (29.9%) of all CEGs were annotated to the ribosome pathway and maintain pregnancy. DEGs were abundant in D30 vs. D20 (3715 DEGs). Besides, the DEGs were associated with the inhibition of oxidative phosphorylation and activation of PI3K-Akt, focal adhesion, ECM–receptor interaction, Rap1, and CAM signaling pathways. The RAP1 may be a central pathway since it coordinates with others to regulate the cell proliferation, invasion, migration, and fusion of trophoblasts. qRT-PCR and Western blot analysis confirmed the transcriptional expression in *IGF1*, *VEGFC*, *RAPGEF3*, *PIK3CA*, *AKT3*, *ITGB3*, *ITGA11*, *SPP1*, *NOS1*, and *ATP6V0B* genes and protein levels in VEGF, RAPGEF3, and Akt. This is the first study of transcriptome profiling in goat placenta and provides diverse genetic resources for further research on placenta development.

## 1. Introduction

The placenta is a unique organ responsible for mammalian viviparity [1,2]. It performs numerous vital functions, including oxygen exchange, transportation of nutrients and waste products, hormone production and regulation, and immunological interaction during gestation [3,4,5]. The placenta is formed by trophoblasts generated during early pregnancy through the proliferation and differentiation of trophoblast stem cells (TSCs) [6,7]. At the blastocyst stage, TSCs differentiate into mononuclear cytotrophoblasts (CTBs) [8,9], maintaining rapid growth and adhesion associated with classical markers including Ki67, CD31, and SPP1 [10,11]. A portion of CTBs, invade the uterine luminal epithelium (LE) to form extravillous trophoblast (EVT). EVT anchors the placenta to the uterine wall and remodels maternal spiral arterioles to guarantee adequate blood supply to the developing fetus [12]. The other CTBs behind EVT are fused into syncytiotrophoblast (STB). STB secretes the requisite hormones for pregnancy maintenance and forms a barrier for nutrient and gas exchange [12,13]. Simultaneously, mesodermally derived precursor cells differentiate into vascular endothelial cells, forming the initial capillaries that follow the direction of CTBs invasion. Besides, they combine with CTBs and STBs to constitute villous trophoblasts (VTS) [14,15]. These complex and delicate placentation events mainly occur between 14 and 30 days of gestation and are associated with embryo loss in domestic animals [16,17]. Thus, early gestation is the most critical period for trophoblast growth and development that determine the success of placentation.

The domestic goat (*Capra hircus*) is one of the most crucial economic livestock. Besides, its successful pregnancy and fetal health safeguard reproductive performance and meat production. The Dazu black goat is a popular breed in southwest China and has strong adaptability and high fecundity [18]. Its natural estrous period can last up to 41.4 h. The average litter sizes of the Dazu black goat (260%) are higher than other goat breeds, such as Matou (214%) and Boer (180%) goats [19]. Therefore, proper placental development in Dazu black goats can produce more lambs than other goat breeds. Candidate genes and signaling pathways in trophoblasts should be screened to explore the mechanisms of early placenta development in Dazu black goats. Such genetic information can provide valuable references for diagnosing placental diseases to indirectly prevent fetus loss. Several studies have reported that ruminant implantation begins with a long pre-attachment period lasting 2–3 weeks [20]. An apposition starts on days 18–20 of pregnancy and establishes cellular contact between the trophoblast and the uterine epithelium [20,21]. Finally, adhesion occurs and produces the cellular structure of an epitheliochorial placenta [22]. Blood vessel cells form in the fetal placenta of sheep on the 24th day of gestation [23]. Ovine trophoblasts continually invade the caruncle deeply with rapid blood vessel growth on the 30th day, before the appearance of the trophoblastic villi [24]. Therefore, placenta development in ruminants depends on trophoblast growth during early pregnancy.

Numerous physiological processes and histological structures have been reported in early placenta development in cattle and sheep [24,25]. However, the goat placenta implantation and development processes have been neglected, mainly referenced from sheep. The lack of known functional genes limits the exploration of the mechanism of placental development in goats. Previously, transcriptome studies through RNA sequencing of trophoblast tissues or cells have been widely applied to examine the potential molecular mechanisms influencing placental development in livestock, including pigs [26], cattle [27], and sheep [28]. Although several functional genes have been further identified from the transcriptome data in these domestic animals, there is little information on the genetic transcriptome of trophoblast in goats.

This study aimed to explore the hircine trophoblast growth and development characteristics on the 20th, 25th, and 30th days of pregnancy in goats. The trophoblast transcriptome corresponding to the different gestation days was also analyzed to screen the common highly expressed genes (ChEGs) and differently expressed genes (DEGs). In addition, crucial signaling pathways related to trophoblast implantation, growth, and angiopoiesis were annotated. Overall, these results provide insights into understanding the molecular genetic mechanism of early placenta development in goat.

## 2. Materials and Methods

### 2.1. Animals, Experimental Design, and Trophoblast Sample Collection

All goat experiments followed the Southwest University Institutional Animal Care and Use Committee (2019, No. GB14925-2010) regulations. In addition, Chongqing Key Laboratory of forage & Herbivore approved the experiments. Nine 10-month-old Dazu black female goats were obtained from the breeding farm at Southwest University. These goats were virgin, unrelated (no common parents), and weighed about 35–40 kg. They were subjected to a standard feeding management regime (DB50/T 510-2013) and housed in free, semi-open folds [18]. The goats were randomly divided into three groups (three replicates per group). Estrus synchronization was conducted in each goat, then mated with a two-year-old Dazu Black buck. The goats were sacrificed on the 20th, 25th, and 30th pregnancy days (D20, D25, and D30), via exposure to carbon dioxide. Trophoblast tissues were then obtained from the gravid uteri. Lastly, the collected trophoblast tissues were either frozen in liquid nitrogen awaiting RNA and protein isolation or fixed in 4% paraformaldehyde (PFA) for subsequent sectioning.

### 2.2. Hematoxylin-Eosin and Immunohistochemistry Staining

Whole trophoblast samples were fixed in 4% PFA for 24 h at room temperature. They were washed thrice with PBS and continuously dehydrated with 50%, 70%, 80%, 90%, and 100% ethanol for 30 min each. After clearing in xylene, the tissues were embedded in paraffin, blocked, and sectioned transversely (6 µm) using a Leica HistoCore BIOCUT microtome (RM2245). The sections were deparaffinized and rehydrated to prepare for hematoxylin-eosin (H&E) and immunohistochemistry (IHC) staining. Next, the slides were soaked in hematoxylin for 15 min and rinsed with running water for H&E staining. After 2 min, all slides were dehydrated in 70%, 95%, and 100% ethanol series for 20 s each. The slides were brightened in xylene for 3 min and covered using the Permount TM Mounting Medium. For IHC staining, the slides were steeped in antigen retrieval solution (10 mM trisodium citrate, 0.05% Tween 20, pH = 6.0) for 30 min at 120 °C. Next, they were incubated in 3% hydrogen peroxide (H_2_O_2_) solution for 45 min at room temperature. After blocking with 3% BSA for 1 h, sections were incubated with primary antibodies overnight at 4 °C and with secondary antibody (HRP) for 1 h at room temperature. The sections were stained with 3,3′-diaminobenzidine (DAB) substrate solution (0.1% DAB, 3% H_2_O_2_, pH = 7.4) for 50 min and counterstained with hematoxylin for 15 min. The slides were dehydrated, as described above. Finally, all HE and IHC section images were captured with Olympus DP73 camera installed on an Olympus IX51 inverted microscope.

### 2.3. RNA Extraction and Quality Controls

Total RNA was extracted from trophoblast tissues using Trizol^®^ reagent following the manufacturer’s instructions (Cat.15596026, Invitrogen, Carlsbad, CA, USA). On the other hand, genomic DNA was removed using DNase I (Takara Biotechnology Co., Beijing, China) treatment. RNA purity and concentration were assessed using the NanoPhotometer^®^ spectrophotometer (IMPLEN, Westlake Village, CA, USA) and Qubit^®^ RNA Assay Kit in Qubit^®^ 2.0 Fluorometer (Life Technologies, Carlsbad, CA, USA), respectively. Lastly, RNA integrity was assessed using the RNA Nano 6000 Assay Kit of the Bioanalyzer 2100 system (Agilent Technologies, Santa Clara, CA, USA). 

### 2.4. Library Preparation for Transcriptome Sequencing and Bioinformatics Analysis

A total of 5 µg RNA per sample was used as input material to generate sequencing libraries using NEBNext^®^ Ultra™ RNA Library Prep Kit for Illumina^®^ (Cat.E7530L, NEB, Ipswich, MA, USA) as recommended by the manufacturer. After purification and fragmentation, mRNA was reverse transcribed into cDNA using the random hexamer primer, M-MuLV Reverse Transcriptase (RNase H), and DNA Polymerase I. The synthesized cDNA fragments were purified with the AMPure XP system (Beckman Coulter, Beverly, CA, USA), and 150–200 bp fragments selected. PCR was performed with Phusion High-Fidelity DNA polymerase, Universal PCR primers, and Index (X) Primer. Finally, PCR products were purified (AMPure XP system), and library quality was assessed using the Agilent Bioanalyzer 2100 system. After sequencing using the Illumina HiSeq 4000 (Majorbio Bio-Pharm Technology Co., Ltd., Shanghai, China) platform, the raw sequence data were filtered with SeqPrep and Sickle software to remove the adapter sequences and low-quality reads with a quality score < 20 or read length < 20 bp. The goat reference genome (ARS1) and gene model annotation files were directly downloaded from the genome website. Lastly, the reference genome index was built using Bowtie v2.2.3, and the clean reads of each sample were aligned to the reference genome using TopHat v2.0.12. 

### 2.5. Gene Expression Level Quantification, Commonly Expressed Genes (CEGs), and Differently Expressed Genes (DEGs) Analysis 

We quantified gene expression level based on the fragments per kilobase of transcript sequence per million base pairs sequenced (FPKM). Every gene was calculated as the expected number of the gene expressed. Subsequently, to determine CEGs, the mean levels (FPKM) of genes were concurrently identified in all groups (*p* > 0.05 or log_2_FC < 1). Lastly, a pairwise comparison among three groups (D20, D25, and D30) was performed to assess the DEGs. The mean level (FPKM) of genes at *p* < 0.05 and log_2_FC > 1 were considered differentially expressed.

### 2.6. GO, KEGG, and PPI Enrichment Analysis

The functional enriched classification analyses, including Gene Ontology (GO) and Kyoto Encyclopedia of Genes and Genomes (KEGG) terms, were used to predict common highly expressed genes (ChEGs) and DEGs using the GOseq R package and KOBAS software (http://kobas.cbi.pku.edu.cn/home.do (accessed on 15 March 2021)). GO and KEGG terms with corrected *p* < 0.05 were defined as significantly enriched by ChEGs and DEGs. A computational method using protein–protein interaction networks (PPI) based on the STRING protein interaction database was performed to analyze the DEGs. Finally, the PPI network image was constructed using Cytoscape v3.4.0 software. 

### 2.7. Quantitative Real-Time PCR (qRT-PCR) for RNA Sequence Validation

Total RNA from trophoblast tissues was extracted as previously described in 2.3. The RNA (2 μg) was reverse transcribed in a 20 μL reaction system using the All-In-One 5×RT MasterMix (Abm, Cat#G592). Next, real-time quantitative PCR (qRT-PCR) was performed using a CFX96 Real-Time System (BIO-RAD) and BlasTaq 2×qPCR MasterMix (Abm, Cat#G891). The gene-specific primers are listed in Supplementary Table S1. The relative mRNA level of all genes was normalized to the expression level of the β-actin gene and calculated using the 2^−ΔΔCt^ method.

### 2.8. Protein Extraction and Western Blotting 

Total protein was isolated from trophoblast tissues using the RIPA buffer (25 mM Tris-HCl (pH 8.0), 150 mM NaCl, 1 mM EDTA, 0.5% NP-40, 0.5% sodium deoxycholate, and 0.1% SDS), phenylmethanesulfonyl fluoride (PMSF), and phosphatase inhibitors, including NaF and Na_3_VO_4_. The protein concentrations were measured using the Pierce BCA Protein Assay Reagent (Pierce Biotechnology) and the NANO drop electrophoresis machine. Proteins were separated through SDS-PAGE and transferred to a polyvinylidene fluoride membrane (PVDF, Millipore Corporation, Burlington, WI, USA). They were blocked in 5% bovine serum albumin (BSA) for 1 h at room temperature, incubated with primary antibodies overnight at 4 °C, and subsequently with secondary antibodies for an hour at room temperature. All primary and secondary antibodies are presented in Supplementary Table S2. Finally, signals of the PVDF membrane were detected using enhanced chemiluminescence Western blotting substrates (Santa Cruz Biotechnology, Shanghai, China) on a Fluor ChemiDoc™ XRS^+^ System (Bio-RAD, Richmond, CA, USA). 

### 2.9. Statistical Analysis

The cluster cross-section size and capillary number density (capillary number per tissue area) of trophoblasts were measured and calculated using the Image J software (NIH, Bethesda, MA, USA). All trophoblast phenotypic data are presented as mean ± SEM (standard error of the mean). One-way ANOVA determined statistical differences at each time point for all the groups in SPSS Statistics version 19.0 (SPSS Inc., Chicago, IL, USA). Lastly, the *p* < 0.05 was considered statistically significant.

## 3. Results

### 3.1. Hircine Trophoblast Showed Significant Growth, Angiogenesis, and Adhesion at Early Pregnancy Stages

**Figure 1:**
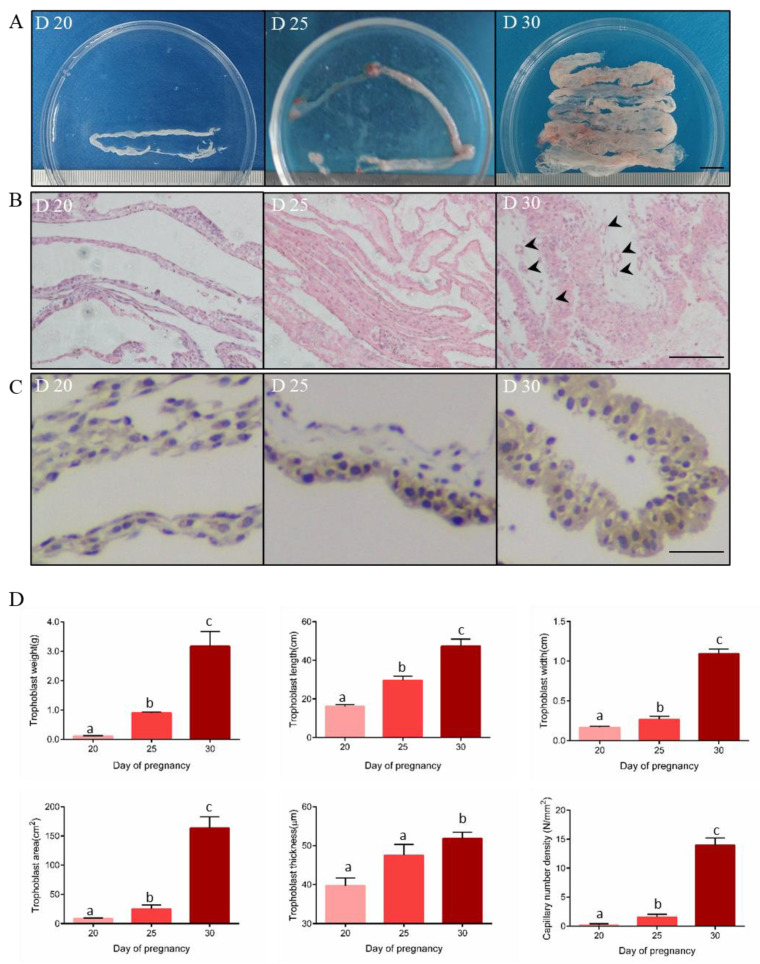
The morphological phenotype and histological structure of trophoblasts on the 20th, 25th, and 30th days of pregnancy (D20, D25, and D30) in Dazu black goats. (**A**). Representative images of trophoblast at the three-time points of early gestation. Scale bar: 1 cm. (**B**). Hematoxylin and eosin (HE) staining of trophoblast at three-time points of early gestation. Scale bar: 200 μm. The black arrow indicates capillary position. (**C**). Immunohistochemical staining of SPP1 on trophoblast at the three-time points of early gestation. Scale bar: 50 μm. (**D**). The average value of trophoblast weight (μm), length (μm), width (μm), area (μm^2^), cross-section thickness (μm) and capillary number density (N/mm^2^). *n* = 3 in each group. Data are expressed as mean ± SEM. Different lowercase letters in the same panel indicate statistical significance (*p* < 0.05).

Due to unknown characteristics in early trophoblast phenotypes of goats, this study first examined the morphology and histological sections of trophoblast on days 20, 25, and 30 in pregnant Dazu black goats. According to the representative images, trophoblasts maintained tubular structures that gradually increased as gestation progressed (Figure 1A). As observed from the 20th to the 30th-day of pregnancy (Supplementary Table S3), the trophoblast membrane sizes significantly (*p* < 0.05) increased from 0.11 ± 0.03 to 2.34 ± 1.08 g (weight), 16.14 ± 1.50 to 47.32 ± 5.16 cm (length), 0.17 ± 0.02 to 1.09 ± 0.08 mm (width), and 8.46 ± 1.47 to 163.11 ± 27.89 cm^2^ (surface area) (Figure 1D). After H&E staining, the trophoblast thickness remained constant between days 20 and 25 of pregnancy (39.63 ± 11.33 μm and 42.74 ± 15.83 μm, respectively). However, it significantly (*p* < 0.05) increased on the 30th day (51.80 ± 10.58 μm) (Figure 1B,D). Meanwhile, the blood capillary near the fetal side was hardly found before 25 days. In contrast, they were present on the 30th day (*p* < 0.05) (Figure 1B), with a capillary number density of 13.93 ± 3.56 N/mm^2^ (Figure 1D and Supplementary Table S3). Subsequently, we detected the expression levels of SPP1 (a molecule marker of trophoblast adhesion) in the trophoblast tissues through IHC staining to observe placenta implantation (Figure 1C). On the 20th day, the SPP1 signal was weak in the cytoplasm and cytomembrane regions of the trophoblast cells. SPP1 expression increased and moved closer to the endometrial caruncular side after 25 days of pregnancy (Figure 1C). On the 30th day, the SPP1 signal was strong in all the trophoblast samples (Figure 1C). These results revealed considerable growth, angiogenesis, and adhesion in the initial goat trophoblast.

### 3.2. Identification of Expressed Transcripts in the Hircine Trophoblast Membrane Transcriptome

All RNA samples were of high quality. The RNA concentration was >10 μg, OD260/280 = 2.0 to 2.3, OD260/230 ≥ 1.9, 28S/18S ≥ 2.2, and RIN > 8.0 (Supplementary Table S4). In each sample, the clean reads from RNA sequencing varied widely from 52,865,644 to 66,566,048 (Supplementary Table S5). The clean reads mean Error rate was maintained at 0.03% in each library. In general, all libraries presented good quality, with an average of 91.76% clean reads with Quality Phred value ≥ 30 (Q30) and 96.77% clean reads with Quality Phred value ≥ 20 (Q20) (Supplementary Table S5). In total, 94.33–94.48% clean reads from each sample were mapped to the hircine reference genome (Supplementary Table S6), containing 1.24–1.75% multiple species and 89.86–91.28% unique maps. The clean reads distribution from all libraries with different regions showed 87.85–92.20% in annotated exons, 3.04–5.48% in introns, and 4.12–6.99% in intergenic regions (Supplementary Table S7). These coverage statistics indicated that the alignment results were appropriate. Hence, the clean reads could be used to enhance further analyses. 

### 3.3. The Top 100 CEGs of Trophoblast Were Highly Related to Protein Translation and Placental Development

**Figure 2:**
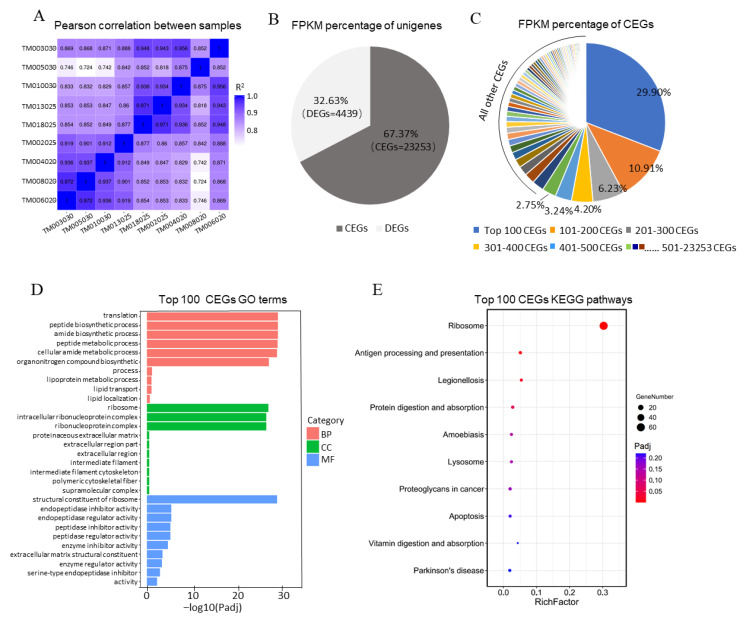
The expression profiling and gene annotation of commonly expressed genes (CEGs) on the 20th, 25th, and 30th days of pregnancy in Dazu Black goats. (**A**). Pearson correlation analysis of all trophoblast samples in response to the three-time points of pregnancy. (**B**). Pie chart showing the distribution of total FPKM in commonly expressed genes (CEGs) and differently expressed genes (DEGs). (**C**). Pie chart showing the distribution of the total FPKM in every 100 CEGs. (**D**). Gene ontology (GO) functional classification of common highly expressed genes (ChEGs) (The top 100 CEGs). BP; biological process, MF; molecular function, CC; cellular component. (**E**). Kyoto encyclopedia of genes and genomes (KEGG) pathway enrich of ChEGs (The top 100 CEGs).

The correlation between samples was assessed using the Pearson correlation method. Except for one D30 sample (No: TM005030), the other samples had medium correlation indexes similar to all the D20 samples, including 0.724, 0.742, and 0.746, respectively. The heatmap showed high correlation indexes, ranging from 0.818 to 0.948 in any two samples with different pregnancy days (Figure 2A). The CEGs and DEGs were further separated and analyzed using the number and mean FPKM percentage. The FPKM expression of every CEG and DEG is illustrated in Supplementary file 1. We found 23,253 CEGs mainly comprised of unigenes that accounted for about 67.37% of total FPKM and only 4439 DEGs with a total FPKM of 32.63% (Figure 2B). In CEGs, the FPKM values of every 100 CEGs were measured and ranked. Moreover, the common highly expressed genes (ChEGs) were discovered in the top 100 CEGs, occupying a total CEGs FPKM of 29.9%. This proportion of FPKM was higher than other CEGs groups (Figure 2C). We categorized the ChEGs into 30 functional groups based on the GO analysis terms and the top ten signalling pathways of KEGG annotation (Figure 2D,E, and Supplementary file 2). In most of the annotated ChEGs, only 61 genes were significantly enriched in the ribosome signalling pathway (*p* < 0.05) (Figure 2E). According to the GO categorization terms, the ChEGs contributed to the structural constituent of the ribosome and enhanced translation (Figure 2D). All ChEGs are summarized in Table 1 to explore their other functions during placental development for the top 100 most expressed. Apart from the 61 ChEGs related to the ribosome, 21 other ChEGs (*COL4A1*/*2*, *COL18A1*, *CTSB*/*L*, Cystatin-C, *PAG-8*, *APOA1*, *TPT1*, *CALR*, Trophoblast Kunitz domain protein 1, Placenta-expressed transcript 1, *SOLD1*, *RACK1*, *ANXA2*, *BSG*, *CLDN4*, *UBB*, *P4HB*, *CD63*, and *LGALS1*) were revealed. They showed significant biological functions in the trophoblast and placenta. Eighteen were unreported, and they could play other functions in the trophoblast or placenta development.

### 3.4. Identifying DEGs on Trophoblast Membranes among the Three-Time Points

**Figure 3:**
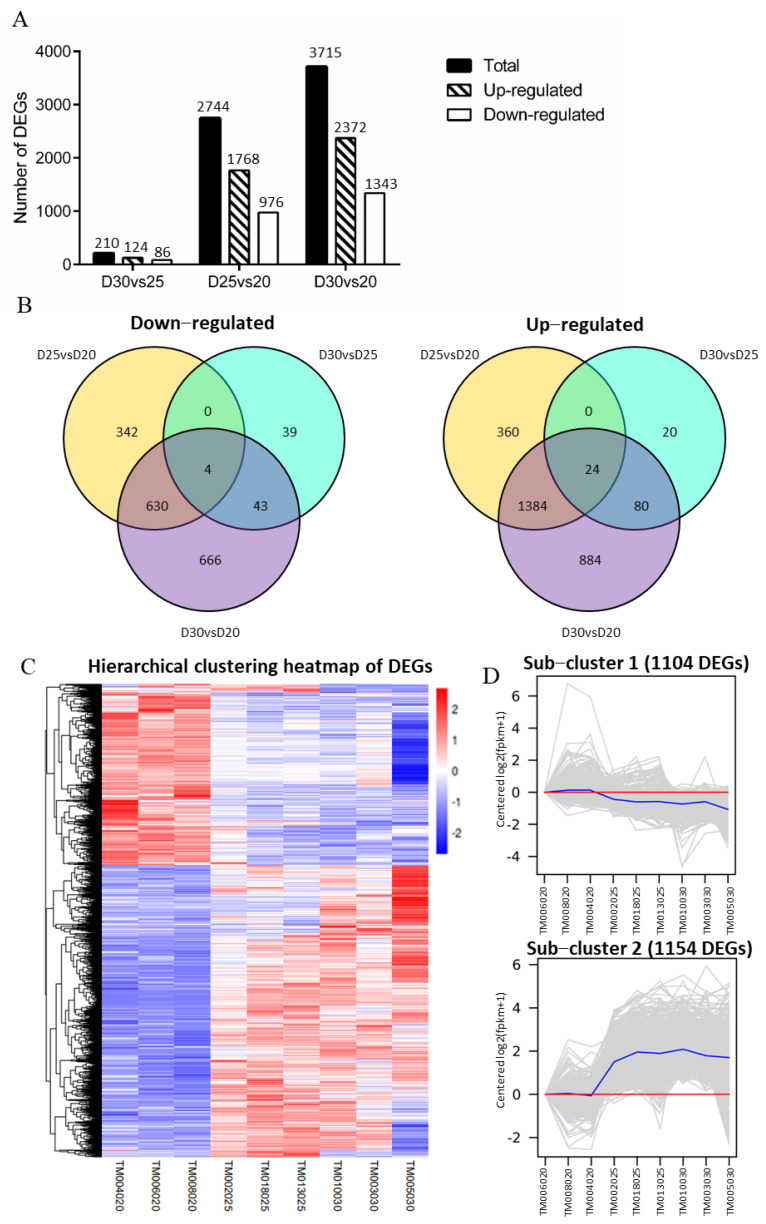
Screening of DEGs in hircine trophoblasts at three-time points of early gestation. (**A**). Quantification of DEGs number in down- and up-regulated portion in the pairwise comparison from three-time points of early pregnancy. (**B**). Venn diagrams illustration of trophoblastic DEGs at three stages of early pregnancy. Left side, down-regulated DGEs. Right site, up-regulated DGEs. (**C**). Hierarchical clustering heatmap of all DEGs performed on nine samples at three-time points of pregnancy. (**D**). The clustering analysis of the expression patterns in down- and up-regulated DEGs.

We performed number and expression trends of DEGs in every pairwise comparison of the three-time points to examine the distribution of DEGs during early pregnancy. Most DEGs were located in D30 vs. D20 (3715) and D25 vs. D20 (2744). In contrast, only 210 DEGs were contained in D30 vs. D25 (Figure 3A). Notably, the number of up-regulated DEGs in all comparison groups was consistently more than that of down-regulated DEGs. There were 2372 up-regulated DEGs and 1343 down-regulated DEGs in D30 vs. D20, 1768 up-regulated DEGs, and 976 down-regulated DEGs in D25 vs. D20, and 124 up-regulated DEGs and 86 down-regulated DEGs in D30 vs. D25 (Figure 3A). Interestingly, the Venn diagrams revealed a few common DEGs between D25 vs. D20 and D30 vs. D25. Both were down-regulated and up-regulated genes, respectively (Figure 3B and Supplementary file 3), suggesting that the expression of all DEGs significantly changed either from D20 to D25 or from D25 to D30. Similarly, the heatmap of all DEGs also presented an increased tendency throughout all the time points (Figure 3C). All DEGs were also enriched in the two main sub-clusters (Supplementary file 4). Generally, 1104 down-regulated DEGs decreased by one-fold of log_2_(FPKM + 1), and 1154 up-regulated DEGs increased by two folds of log_2_(FPKM + 1), and the expression change of all DEGs mainly occurred on the 25th day of pregnancy (Figure 3D).

### 3.5. Analysis of Functional Annotation and Pathway Enrichment in the Main Clusters of DEGs

**Figure 4:**
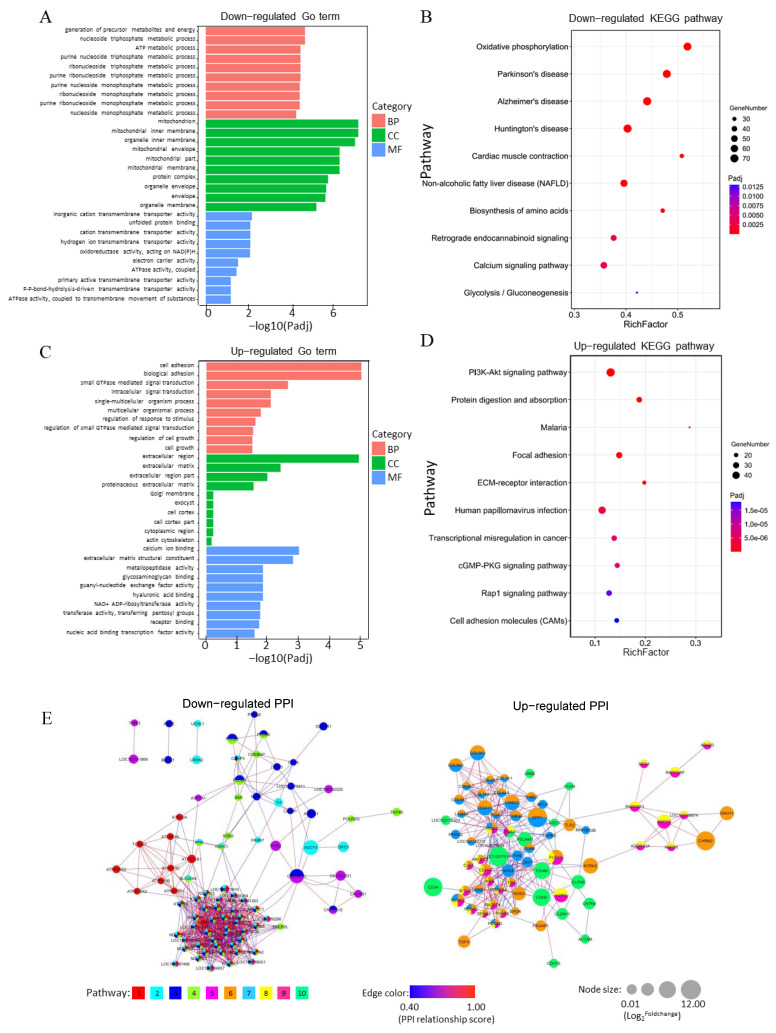
Functional categorization and signaling pathways enrichment of two-cluster DEGs by GO and KEGG classification in trophoblasts at early pregnancy. (**A**). GO functional classification of down-regulated DEGs. BP, biological process; MF, molecular function; CC, cellular component. (**B**). KEGG pathway enrichment analysis of down-regulated DEGs. (**C**). GO functional classification of up-regulated DEGs. BP, biological process; MF, molecular function; CC, cellular component. (**D**). KEGG pathway enrichment analysis of up-regulated DEGs. (**E**). Protein-protein interaction (PPI) analysis of two-cluster DEGs at three early pregnancy stages. Left side, down-regulated DGEs. The right site indicates up-regulated DGEs. Pathway: 1, Oxidative phosphorylation. 2, Parkinson’s disease. 3, Alzheimer’s disease. 4, Huntington’s disease. 5, Cardiac muscle contraction. 6, PI3K-Akt signaling pathway. 7, Focal adhesion. 8, ECM-receptor interaction. 9, Rap1 signaling pathway. 10, Cell adhesion molecules (CAMs).

We annotated two-cluster DEGs from the GO and KEGG pathway analyses as the up-regulated and down-regulated DEG clusters, respectively. In the down-regulated cluster, DEGs related to the major GO terms caused significant differences. They generated precursor metabolites and energy during biological processes, mitochondrion in cellular components, and inner mitochondrial membrane protein complex in molecular functions (Figure 4A and Supplementary file 5). In the KEGG pathway analysis, 17 pathways were significantly altered (Supplementary file 6). The top five significant pathways were mainly attributed to oxidative phosphorylation, Parkinson’s disease, Alzheimer’s disease, Huntington’s disease, and cardiac muscle contraction (Figure 4B). In the up-regulated cluster, DEGs were enriched in the extracellular region of cellular components, cell adhesion of biological processes, and aspartic-type endopeptidase activity on molecular functions by GO terms (Figure 4C and Supplementary file 7). Meanwhile, 36 pathways were significantly changed in KEGG analysis (Supplementary file 8). Some potential pathways, including PI3K-Akt, focal adhesion, ECM-receptor interaction, Rap1, and CAM, were involved in placenta development (Figure 4C). The predicted interaction networks on the down-regulated DEGs participated in oxidative phosphorylation, Parkinson’s disease, Alzheimer’s disease, Huntington’s disease, and cardiac muscle contraction pathways. On the other hand, those on up-regulated DEGs were associated with PI3K-Akt, focal adhesion, ECM–receptor interaction, cGMP-PKG, Rap1, and cell adhesion molecule pathways (Figure 4D).

### 3.6. Rap1 Signaling Pathway Connects with Other Functional Pathways to Regulate Placental Development

**Figure 5:**
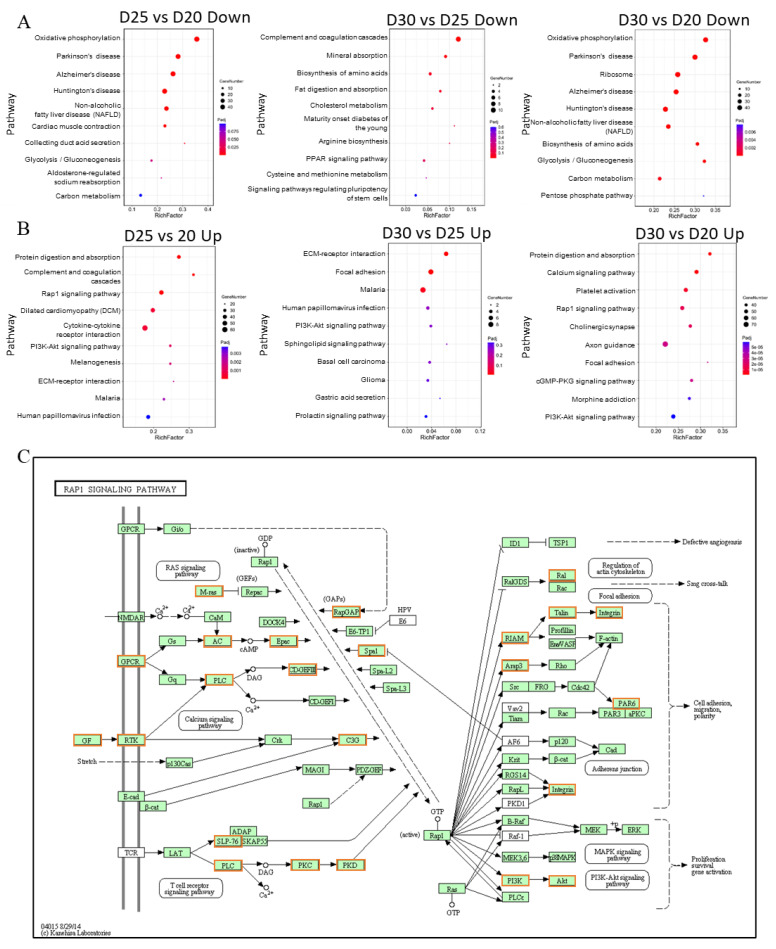
Signaling pathways enrichment of down- and up-regulated DEGs based on KEGG in the pairwise comparison from three-time points of pregnancy. (**A**). KEGG pathway enrichment of down-regulated DEGs in D25 vs. D20, D30 vs. D25, and D30 vs. D20. (**B**). KEGG pathway enrichment of up-regulated DEGs in D25 vs. D20, D30 vs. D25, and D30 vs. D20. (**C**). Distribution of special DEGs in Rap1 pathway signaling pathway. The red frame indicates up-regulated DEGs.

We checked annotated signaling pathways in all down- and up-regulated DEGs using pairwise comparison of three pregnant time points. Similarly, oxidative phosphorylation and neuro-related disease signaling pathways were significantly down-regulated in D25 vs. D20 and D30 vs. D20 (Figure 5A). In particular, the complement and coagulation cascade pathways were significantly down-regulated in D30 vs. D25 (Figure 5A). In the up-regulated groups, Rap1, PI3K-Akt, focal adhesion, ECM–receptor interaction, and cytokine-cytokine receptor interaction signaling pathways changed significantly in the top 10 signaling pathways in D25 vs. D20 and D30 vs. D20 (Figure 5B). After analyzing these significant pathways in KEGG, the PI3K-Akt, focal adhesion, and calcium signaling pathways were related to the Rap1 signaling pathway, regulating cell proliferation, migration, and invasion (Figure 5C). In D30 vs. D20, there were 54 up-regulated DEGs with red frames annotated in the Rap1 signaling pathway. They included growth factor genes (*KITLG, IGF1, VEGFA, VEGFC, EFNA2, PDGFD, PDGFC, PDGFRA, FGF13, FGFR4, ANGPT4, ANGPT2, CSF1, CSF1R,* and *INSR*), adenylate cyclase (*ADCY2, ADCY4, ADCY5,* and *ADCY6*), integrins (*ITGA2B, ITGAM, ITGB2*, and *ITGB3*), phosphoinositide-3-kinases (*PIK3R1* and *PIK3CD*), phospholipase C (*PLCB1, PLCB2, PLCB4,* and *PLCG1*), Rap guanine nucleotide exchange factors (*RAPGEF3, RAPGEF4, RAPGEF1, Rap1GAP, ARAP3, SIPA1,* and *APBB1IP*), RAS-related guanyl releasing proteins (*RASGRP3, PARD6G, RALB,* and *MRAS*), protein kinases (*PRKCB, PRKD1,* and *PRKD3*), tyrosine kinases (*FLT4* and *TEK*), adenosine receptors (*ADORA2B* and *ADORA2A*), lymphocyte cytosolic protein 2 (*LCP2*), AKT serine/threonine kinase 3 (*Akt3*), F2R Like Thrombin/Trypsin Receptor 3 (*F2RL3*), Talin 2 (*TLN2*), *LOC108638200*, *LOC102190430*, and *LOC102188874* (Supplementary file 9).

### 3.7. Validation of DEGs Expression and Summary of Physiological Processes in the Trophoblast Membrane of Goats

**Figure 6:**
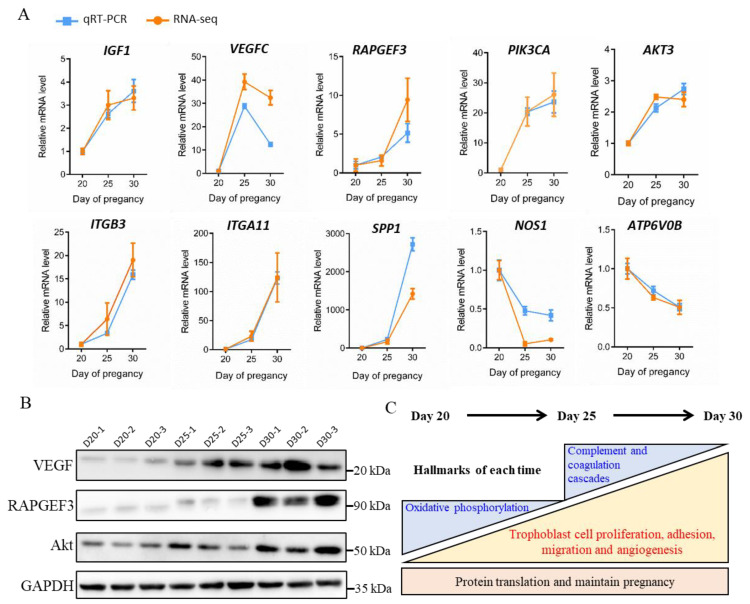
Validation of DEGs expression and summary of physiological processes at early placenta development in goats. (**A**). Comparison of gene expression levels between RNA-Seq and qRT-PCR in trophoblast at 20, 25, and 30 days of pregnancy. (**B**). Western blot showing the translational expression level of candidate genes (VEGF, RAPGEF3, and Akt) in trophoblast at 20, 25, and 30 days of pregnancy. (**C**). Summary diagram showing the functional annotations for ChEGs and DEGs in trophoblast by the time of placenta development.

The early placenta development-induced genetic changes were further confirmed using qRT-PCR and western blot analysis. Ten DEGs, including *IGF1*, *VEGFC*, *RAPGEF3*, *PIK3CA*, *Akt3*, *ITGB3*, *ITGA11*, *SPP1*, *NOS1*, and *ATP6V0B*, were selected from Rap1, oxidative phosphorylation, and Alzheimer’s disease signaling pathways. Their transcriptional level was measured through qRT-PCR (Figure 6A). Consequently, the protein level of VEGF, RAPGEF3, and Akt was further verified using the Western blot technique (Figure 6B). All qRT-PCR and western blot findings were consistent with those obtained from previous sequence analysis. Finally, we summarized this study in a diagram describing functional annotations of CEGs and DEGs in trophoblasts during early pregnancy stages (Figure 6C). During the hircine placenta development from the 20th to the 30th day of pregnancy, the CEGs were located in the base rectangle, showed high protein translation expression, and maintained pregnancy. The up-regulated DEGs in the middle region of the big triangle mediated key pathways to promote trophoblast proliferation, adhesion, migration, and angiogenesis. Finally, the down-regulated DEGs were distributed on the two small triangles in the top region with reduced oxidative phosphorylation and complement and coagulation cascades.

## 4. Discussion

In mammals, the placenta is the only gestational organ with vital functions of oxygen exchange, nutrient supply, hormone regulation, and immunological interaction during pregnancy [3,4,5]. The placenta originates from the trophoblast at the blastocyst stage. The growth and development of placenta characterized by trophoblast growth, differentiation, implantation, villi formation, and angiopoiesis [15,24]. Besides, these processes are closely related to pregnancy maintenance, embryonic loss, and even lamb health [16,17]. The domestic goat (*Capra hircus*) is one of the most crucial economic livestock, whose normal placenta development safeguards lamb littering, health, and meat production [18]. Therefore, investigating tissue structure and screening potential functional genes in trophoblasts are necessary to explore the molecular mechanisms of placenta formation at the early pregnant stage. In this study, the morphology and histological structures of trophoblasts showed significant growth, adhesion, and angiogenesis. The ChEGs with the highest FPKM percentage (29.9%) of all CEGs were annotated to ribosome pathway and maintain pregnancy. Besides, the DEGs were associated with the inhibition of oxidative phosphorylation and activation of PI3K-Akt, focal adhesion, ECM–receptor interaction, Rap1, and CAM signaling pathways.

We found that the 20th, 25th, and 30th days of pregnancy could be the critical time points for trophoblast cell proliferation, adhesion, and angiogenesis in goats. In our study, the weight, length, width, and area of hircine trophoblast showed a continuous and notable increase in from the 20th to 30th day of pregnancy, similar to previous reports in sheep [23,24]. Ovine trophoblast thickness showed a significant increase, starting at the 24th day after fertilization [23], but hircine trophoblast thickness significantly increased at the 30th pregnant day, which suggested a five-day delay of trophoblast growth in goat. Meanwhile, we found that trophoblast length increased earlier than the trophoblast cell layers. This imbalance rate of hircine trophoblast growth, between perpendicular and parallel to the endometrium, suggested a growth characteristic of firstly placenta elongation, and then trophoblast thickened during early pregnancy. The new blood vessels emerged in the trophoblast at 25 days post-pregnancy and were widely generated on day 30 of the pregnancy, consistent with those in sheep [24]. Trophoblast cell adhesion is the last step of implantation in ruminants, after attachment and apposition [20]. SPP1 (secreted phosphoprotein 1) is a leading candidate adhesion protein for implantation in pigs and sheep [11,56]. Our results showed that SPP1 was hardly expressed on the 20th day of pregnancy but considerably expressed on the 25th day of pregnancy, suggesting the beginning and increase of trophoblast adhesion. Thus, our results confirmed the fundamental development process of trophoblast in Chinese indigenous goats.

The common highly expressed genes (ChEGs) may participate in protein translation and maintain pregnancy at the early pregnancy. The commonly expressed genes (CEGs) in tissues at different time periods have been reported to have critical foundational functions related to their organizational characteristics [57,58]. In our study, the short interval (five days) of sampling in trophoblast tissues suggested a similar basic biological process of placentation. Since the high-expressed portion of CEGs, also named ChEGs, are associated with tissue-specific physiological activities [59]. Thus, we selected the top 100 CEGs as the ChEGs for bioinformatics analysis. Over 60% of the ChEGs were ribosomal genes, of which some (RPL26 and RPL10) are involved in ribosome assembly and protein synthesis in primary human trophoblast cells [29]. Moreover, the other 21 ChEGs were reported from previous studies and are essential to regulation functions in placental development. For example, *CTSB* (*Cathepsin B*) can regulate the trophoblast cell migration, apposition, and remodeled placenta [32], LOC102184534 (Cystatin-C) specifically expressed in extravillous trophoblast and regulated protease activity in placentation [34], and *PAG8* (Pregnancy-associated glycoprotein-8) gene can maintain pregnancy at different gestation stages in Barbari goats [29]. Furthermore, the remaining 18 genes from ChEGs have not been reported in the trophoblast or placenta. Hence, it is imperative to explore the new functions of these genes in placental development.

The down-regulated DEGs of trophoblast during early pregnancy were regulated by uterine hypoxic environment and maternal-fetal receptivity. In D25 vs. D20, the down-regulated DEGs were significantly enriched in oxidative phosphorylation. It suggested the low oxygen consumption and inhibition of mitochondrial function in early pregnancy [12,60]. Previous studies have reported that placental development is initiated with low intervillous oxygen tension, i.e., 2–3% O_2_, which is significantly lower than endometrial oxygen tension [12]. Under such low oxygen levels, oxidative phosphorylation of mitochondria was inhibited in the trophoblast [61]. However, hypoxic conditions are necessary to EVT (extravillous trophoblast) lineage commitment and the survival of EVT progenitor cells through the hypoxia-inducible factor (HIF) pathway [62]. Herein, HIF3α was found as the only member of the HIF family that was significantly increased in goat trophoblast (Supplementary file 2). And it promotes EVT growth via activation of Flt1-JAK-STAT signaling pathway in hypoxia [63]. As oxygen tensions rise in the first trimester later, EVT progenitors mature, increasing invasive EVTs, which further invade and remodel maternal spiral arterioles [62,64]. In addition, HIF is an important component of vasculature formation by synergistic correlations with other proangiogenic factors, such as VEGF (vascular endothelial growth factor) and PlGF (placental growth factor) [65]. Our study showed capillaries appeared in the 30th day of pregnancy by HE staining and the decrease of oxidative phosphorylation was suspended from D25 to D30, indicating that the blood carries oxygen to the trophoblastic tissue, thereby inhibiting the further decrease of oxidative phosphorylation and promoting EVT invasion to the endometrium.

The down-regulated DEGs in D30 vs. D25 were annotated to the complement and coagulation cascades, which is a major component of innate immunity in the blood associated with the mechanisms of fetal loss and placental inflammation in the antiphospholipid antibody syndrome [66]. Previous studies have demonstrated that physiologic complement activation facilitates the clearance of fetoplacental debris and protects against pathogens in normal pregnancy [67,68]. Moreover, women with increased complement activation in early pregnancy are more likely to develop preeclampsia and adverse pregnancy outcomes [69]. Therefore, the inhibition of the complement system promotes a healthy placenta at early pregnancy in goats.

The up-regulated DEGs of trophoblast during early pregnancy were involved in placenta cell proliferation, adhesion, and angiopoiesis. Our results have shown that the up-regulated DEGs are significantly enriched to PI3K-Akt, focal adhesion, ECM–receptor interaction, Rap1, and CAMs signaling pathways through the KEGG term analysis. Focal adhesion, ECM–receptor interaction, and CAMs are the classical cell adhesion pathways [70,71]. Focal adhesions are integrin-containing, multi-protein structures, providing signaling links between the intracellular actin cytoskeleton and extracellular matrix in many cell types [71]. They function by transmitting force at cell adhesion sites and serve as signaling centers from which numerous intracellular pathways can regulate cell growth, proliferation, survival, development, tissue repair, migration, and invasion [72]. ECM integrin-binding at this interface provides high tensile forces and signaling to coordinate uteroplacental development during sheep implantation [73]. This results in a focal adhesion containing integrin aggregates and ECM protein in LE and EVT [74]. This study is consistent with the activation of trophoblast (ITGA11 and ITGB3) to form robust focal adhesions in response to binding ligands associated with the apical surfaces of these cells. We present direct in vivo evidence showing functional roles of integrins in signaling concepts and maternal tissues in early stages of pregnancy.

There was an indication that PI3K-Akt pathway could be involved in the growth and angiogenesis of trophoblast. The phosphatidylinositol 3-kinase/protein kinase B (PI3K-Akt) pathway is frequently activated in various cells and promote numerous cellular functions, including proliferation, adhesion, migration, invasion, and angiogenesis [75,76,77]. In human trophoblast cells (HTR-8/SVneo), inhibiting phosphorylation in the PI3K-Akt pathway can reduce the phosphorylated endothelial nitric oxide synthase (eNOS/NOS3) and mTOR expression, which markedly decreases cell proliferation, migration, and invasion [76,78]. On the contrary, increasing phosphorylation in the PI3K-Akt pathway regulates cell cycle progression by promoting cells into the S-phase [75]. In recent years, researches have proven the angiogenesis function of the PI3K-Akt pathway in the vascular endothelial cells through *VEGF*-related genes [77,79]. Our study established that the DEGs (*NOS3* and *VEGFC)* enriched in the PI3K/Akt pathway may involve in placentation.

The Rap1 signaling pathway may be a central connection that regulates placenta development by linking upstream growth factors and downstream PI3K/Akt and focal adhesion pathways. Ras-associated protein 1 (Rap1) is a small GTPase belonging to the Ras family of GTPase [80]. It is activated by several Rap1-related guanine nucleotide exchange factors (RAPGEFs) and Ras-related guanyl releasing proteins (RASGRPs) to influence cell proliferation, adhesion, and fusion [81,82,83]. Herein, the mRNA level of *RAPGEF1*, *RAPGEF3*, *RAPGEF4,* and *RASGRP3* significantly increased in trophoblasts from the 20th to 30th pregnancy days. Particularly, RAPGEF3 has been reported to promote BeWo cells fusion via Rap1/CaMKI/HDAC5 signaling cascade [83]. Collectively, this result suggests that RAPGEFs and RASGRPs have multiple potential functions in hircine trophoblast cells. Meanwhile, other studies have shown that Rap1 is activated in response to upstream signaling growth factors that act on receptor tyrosine kinases and G-protein coupled receptors, such as IGF1 and EGF [84,85]. In our study, *IGF1* and *FGF13* gene were identified as the up-regulated DEGs, suggesting activity in the Rap1 signaling pathway in placenta development. Moreover, our findings revealed that the annotated Rap1 signaling pathway presents a link to downstream signaling pathways, PI3K/Akt and focal adhesion. Similar reports have been outlined in regulating the PI3K/Akt pathway in endothelial cells. RASGRP2 activates the Rap1 signaling and suppresses apoptosis by activating the PI3K-Akt signaling pathway [86]. Meanwhile, Rap1 triggers integrin-mediated adhesion of the ECM and regulates the talin recruitment to the β-integrin tail to activate integrin and promote cell spreading, migration, and invasion [87,88]. Besides, RAP1 promotes VEGFR2 activation and angiogenesis in endothelial cells via the integrins [89]. Therefore, the Rap1 pathway could be the most vital multifunctional regulator for trophoblast cell proliferation, migration, invasion, and angiogenesis in the hircine placenta.

## 5. Conclusions

This is the first study to describe trophoblast morphology and transcriptome profiling at the early stage of pregnancy in goats. CEGs and DEGs have been annotated to maintain biological pregnancy processes, trophoblast cell proliferation, migration, adhesion, and angiopoiesis. Up-regulated DEGs annotate the Rap1 pathway, suggesting that it combines with PI3K-Akt and focal adhesion pathways to regulate placental development and implantation. These candidate genes and enriched signaling pathways could be valuable references for exploring the molecular mechanisms underlying ruminant placentation.

## Figures and Tables

**Table 1:** The information of top 100 CEGs related to placenta development at early pregnancy.

Gene Category	Gene Number	Count of PFKM	Functional Description	Reference
RPS (Ribosomal protein S2, 3, 5, 6, 7, 8, 10, 11, 12, 14,15,16, 17, 18, 19, 20, 23, 25, 26, 27, 28, 29, 15A, 27A, 4X), RPL (Ribosomal protein L3, 4, 5, 6, 7, 8, 10, 11, 12, 13, 14, 18, 19, 21, 23, 24, 26, 27, 28, 30, 31, 32, 35, 37, 7A, 13A, 18A, 23A, 27A, 35A, P0, P1), LOC102175427 (40S ribosomal protein SA), LOC102186381 (60S ribosomal protein L34), LOC100861018 (60S ribosomal protein L17), FAU(2C ubiquitin like and ribosomal protein S30 fusion),	61	57,458.1 (Total)	Ribosomal genes related to placental development	[29]
LOC102177175 (Spleen trypsin inhibitor I), RBP4 (Retinol binding protein), FTH1 (Ferritin heavy chain 1), ACTG1 (Actin gamma 1), SECTM1 (Secreted and transmembrane 1), EEF1A1/G (Eukaryotic translation elongation factor 1 alpha 1/gamma), LOC102178813 (ADP/ATP translocase 3), TMSB4X (Thymosin beta 4 X-linked), SERF2 (Small EDRK-rich factor 2), MYL6 (Myosin light chain 6), LOC102182127 (Clusterin), REXO2 (RNA exonuclease 2), LOC102188434(Cytochrome c oxidase subunit 4 isoform 1), SERPINH1(Serpin family H member 1), PPIB (Peptidylprolyl isomerase B), HSPA8/B1(Heat shock protein family A8/B1)	18	17,987.5 (Total)	Unreported genes in trophoblast transcriptomes	/
COL4A1/2, COL18A1 (collagen type IV alpha 1/2 and XVIII alpha 1)	3	708.6/652.8/626.7	Closely associated with extracellular matrix (ECM) production in placenta	[30,31]
CTSB/L (Cathepsin B/L)	2	1750.5/502.5	Regulated the cell migration, apposition, and remodeled placenta for transport of gases, micronutrients, and macromolecules	[32,33]
LOC102184534 (Cystatin-C)	1	1889.1	Highly expressed in the extravillous trophoblast cells of the basal plate and regulated proteases in placentation.	[32]
PAG-8 (Pregnancy-associated glycoprotein-8)	1	1832.6	Maintenance pregnancy	[34]
APOA1 (apolipoprotein A1)	1	1483.6	Resisted to atherosclerosis and kept cholesterol homeostasis between maternal and fetal	[35]
TPT1 (translationally controlled tumor protein 1)	1	1345.3	Regulated calcium handling transport in trophoblast cells	[36]
CALR (Calreticulin)	1	1214.6	Involved in the invasion of extravillous trophoblasts and syncytialization of villous trophoblasts	[37,38]
LOC102188515 (Trophoblast Kunitz domain protein 1-like)	1	1057.3	Participated in maternal recognition of pregnancy in ruminants	[39,40]
LOC102177258 (Placenta-expressed transcript 1 protein)	1	1013.7	Played a key role in the establishment of a stable trophoblast and endometrial epithelial layers	[41]
SOLD1(secreted protein of Ly-6 domain 1)	1	984.15	Regulated cell invasiveness, placental construction, and development of cotyledonary villi	[42,43,44]
RACK1 (receptor for activated C kinase 1)	1	861.1	Involved in migration and invasion activities in human trophoblast BeWo cell	[45]
ANXA2 (annexin A2)	1	802.1	Impaired decidualization of endometrial stromal cells and promoted embryo implantation and placentation	[46]
BSG (Basigin)	1	745.9	Regulated glycolytic flux to promote cells proliferation in embryo implantation and placental development	[47,48]
CLDN4 (Claudin 4)	1	701.5	Located in multinucleated syncytiotrophoblast layer and participate in modulating trophoblast mobility	[49,50]
UBB (Ubiquitin B)	1	608.2	Involved in the degradation of the extracellular matrix (ECM) and trophoblastic invasion during early pregnancy	[51]
P4HB (Prolyl 4-hydroxylase subunit beta)	1	592.2	Predominantly expressed in human extravillous trophoblasts	[52]
CD63	1	575.1	Highly expressed in mesenchymal stem cell exosomes in placenta isolated exosomes expressed the exosome markers	[53]
LGALS1(Galectin 1)	1	531.7	Promoted mouse trophoblast stem cell differentiation and potentiates trophoblast fusion in humans	[54,55]

## Supplementary Materials

The following are available online at https://www.mdpi.com/article/10.3390/ani11072132/s1, Table S1: The information of primers used for genes expression analysis by qRT-PCR; Table S2: Antibody used for immunofluorescence (IHC) and western blot (WB); Table S3: The phonotypes result in trophoblast in goat at three-time points of early pregnancy; Table S4: The quantity and quality of mRNA samples from trophoblast for RNA sequencing; Table S5: The quality of reads from mRNA samples in every trophoblast; Table S6. The quality reads from mRNA samples in every trophoblast; Table S7. The distribution of reads on DNA sequences in every trophoblast.
